# A comparison of the Antileukaemic Effects of Recombinant Human Tumour Necrosis Factor-α and its Muteins on Leukaemia L1210 and Leukaemia P388 in Mice

**DOI:** 10.1155/S0962935194000578

**Published:** 1994

**Authors:** K. Warzocha, J. Góra-Tybor, M. Kwinkowski, B. Szymańska, T. Robak

**Affiliations:** 1 2nd Clinic of Internal Medicine Medical University of Łódź Pabianicka 62 Łódź 93-513 Poland; 2 Department of Pharmacology Medical University of Łódź Muszyńskiego 1 Łódź 90-151 Poland; 3 Department of Bioorganic Chemistry Polish Academy of Sciences Sienkiewicza 112 Łódź 90-363 Poland

## Abstract

We investigated the influence of recombinant human tumour necrosis factor alpha (TNF-α) and its derivatives termed muteins III, V, VI—in which the first 3 to 7 amino acids of native TNF-α have been replaced—on the survival time of mice inoculated with leukaemia L1210 or leukaemia P338. TNF-α prolonged the survival of mice with leukaemia L1210 but did not have any therapeutic activity in leukaemia P388-bearing mice. Muteins-treated mice with leukaemia P388 lived longer than animals receiving TNF-α, while those inoculated with leukaemia L1210 did not show any significant prolongation of life compared with the TNF-α treated group. The results presented in this report indicate that the antileukaemic activity of TNF-α is governed at least in part by the nature of the N-terminal amino acids.


Mediators of Inflammation 3, 411–414 (1994)

### A comparison of the antileukaemic effects of recombinant human tumour necrosis factor-α and its muteins on leukaemia L1210 and leukaemia P388 in mice

K. Warzocha,[1] J. Góra-Tybor,[2] M. Kwinkowski,[3] B. Szymańska[3] and T. Robak[1,CA]

[1]2nd Clinic of Internal Medicine, Medical University of Łódź, Pabianicka 62, 93-513-Łódź, Poland;

[2]Department of Pharmacology, Medical University of Łódź, Muszyńskiego 1, 90-151 Łódź, Poland;

[3]Department of Bioorganic Chemistry, Polish Academy of Sciences, Sienkiewicza 112, 90-363 Łódź, Poland

[CA] Corresponding Author

**We investigated the influence of recombinant human tumour necrosis factor alpha (TNF-α) and its derivatives termed muteins III, V, VI—in which the first 3 to 7 amino acids of native TNF-α have been replaced—on the survival time of mice inoculated with leukaemia L1210 or leukaemia P338. TNF-α prolonged the survival of mice with leukaemia L1210 but did not have any therapeutic activity in leukaemia P388-bearing mice. Muteins-treated mice with leukaemia P388 lived longer than animals receiving TNF-α, while those inoculated with leukaemia L1210 did not show any significant prolongation of life compared with the TNF-α treated group. The results presented in this report indicate that the antileukaemic activity of TNF-α is governed at least in part by the nature of the N-terminal amino acids.**

**Key words:** Experimental leukaemias, Muteins, Tumour necrosis factor

### Introduction

The multifunctional cytokine tumour necrosis factor-α (TNF-α) plays a role in the regulation of many biological responses *in vivo*, and has been implicated in a wide range of pathological conditions, including the host response to leukaemia growth.[1–5] As for cytokines in general, the first event in triggering a cellular response is a specific high affinity interaction with membrane receptor molecules initiating a cascade of signal transfer reactions inside the cell. However, although two cell surface receptors for TNF-α have been identified, the amino acid residues necessary for the biological activity of TNF-α have not been characterized. Experiments performed with derivatives of TNF-α termed muteins, in which the first 3 to 7 amino acids of native TNF-α have been replaced, indicate that the receptor-binding domain of TNF-α may be located near the N-terminus of the molecule.[6,7] In the present study we compare the antitumour effects of TNF-α and its N-terminal region muteins in murine leukaemia model.

### Materials and Methods

*Animals*: All the mice used in this experiment were produced at the Institute of Immunology and Experimental Therapy, Polish Academy of Sciences, Wrocław. At the time of initiation of the experiment they were young adults, 8–12 weeks old. They were given standard laboratory food and water *ad libitum*. In this study, female mice of DBA/2 strain were used.

*Leukaemias*: Leukaemia L1210 and P388 were obtained from the Institute of Immunology and Experimental Therapy, Polish Academy of Sciences, Wrocław, and were maintained by serial passage in the ascitic fluid of DBA/2 mice. Leukaemic cells from the fluid were resuspended in 0.9% sodium chloride such that $10^6$ leukaemia L1210 or P388 cells were injected i.p. into DBA/2 recipients.

*Cytokines*: TNF-α and its muteins' syntheses and biochemical analyses were performed in the Department of Bioorganic Chemistry, Łódź, Poland. Production of TNF muteins were supported by the Committee for Scientific Research Grant No. 662889203 to M.K. and B.S. Recombinant human TNF-α had a specific activity of $2 \times 10^7$ U/mg. Muteins III, V, VI were constructed using synthetic oligonucleotides to introduce changes in the DNA sequence, encoding the 7 N-terminal amino acids of native TNF-α.[8] The DNA was expressed in *Escherichia coli*, and the resulting muteins were purified by ion exchange chromatography. Amino acid sequences were analysed by automated Edman degradation using an Applied Biosystems ABI 477A protein sequencer. The N-terminal amino acids sequences of TNF-α and its muteins are shown in Table 1. Endotoxin contamination amounted to approximately 1.9 ng endotoxin per mg protein, as estimated using a commercially available assay (Sigma Chemical Co., St Louis, MI, USA). Recombinant cytokines were reconstituted using sterile phosphate-buffered saline (PBS), and premixed at a concentration such that all doses were injected in 0.2 ml.

© 1994 Rapid Communications of Oxford Ltd

**Table 1.** The N-terminal amino acids sequences of TNF-α and its muteins

| | |
|---|---|
| TNF-α | Val–Arg–Ser–Ser–Ser–Arg–Thr– |
| Mutein III | Arg–His–Arg–His– |
| Mutein V | Val–Arg–Ser–Ser–Ile–Val–Ile– |
| Mutein VI | Met–Arg–Ile–Arg–Met– |

Met–methionine; Val–valine; Arg–arginine; Ser–serine; Thr–threonine; Lys–lysine; His–histidine; Ile–isoleucine.

*Treatment*: Animals received tumour challenges on day 0, and all treatments were initiated i.p. on the following day. Cytokines were administered at doses of 250 and 400 μg/kg as daily injections for 8 days or at a dose of 400 μg/kg given 2, 4, 6 and 8 days after the inoculation of leukaemic cells. The control group of mice received i.p. injections of PBS.

*Antileukaemic assay*: Animals were observed daily for survival for a minimum of 60 days. The median survival time (MST) was assessed as follows: MST = $(x + y)/2$, where '$x$' denotes the earliest day when the number of dead animals > $n/2$, '$y$' denotes the earliest day when the number of dead animals > $(n/2) + 1$, and '$n$' denotes the number of animals in that group. Therapy efficacy against leukaemia was assessed as a percentage of median survival time of the treated group (T) to that of the control group (C): $T/C = MST_T/MST_C \times 100$.

*Statistical analysis*: Statistical analysis was performed using Student's $t$-test. Results were considered significant when the $p$ value was $\leq 0.05$.

### Results

Different treatment regimes with TNF-α or its muteins III, V, VI against the two types of murine leukaemias were studied to compare their antitumour activities. As shown in Table 2, TNF-α prolonged survival of leukaemia L1210-bearing mice when given daily at a dose of 250 μg/kg ($0.01 > p > 0.001$), but shortened it when given at a dose of 400 μg/kg ($p < 0.001$). Sequential application of TNF-α at a dose of 400 μg/kg did not significantly change the lifetime of mice with leukaemia L1210, but was better tolerated than the same dose applied in a daily treatment regime ($0.01 > p > 0.001$) (Table 3). Conversely, TNF-α muteins did not show any therapeutic activity against leukaemia L1210 when used daily at a dose of 250 μg/kg, but had a comparable therapeutic effect, except mutein III to native TNF-α, when used at a dose of 400 μg/kg, either in daily or sequential injections ($0.01 > p > 0.001$) (Tables 2 and 3). There were no significant changes in lifetime, either for daily or for sequential application, between the groups of mice receiving different TNF-α muteins (Tables 2 and 3).

**Table 2.** The influence of TNF-α and its muteins, administered daily, on the survival time of mice with leukaemia L1210

| Therapeutics[a] | | MST[c] (days) | $\bar{X} \pm SD$[d] | T/C[e] |
|---|---|---|---|---|
| Cytokine | Dose (μg/kg) | | | |
| None | PBS[b] | 7.0 | 6.8 ± 0.5 | |
| TNF-α | 250 | 10.5* | 10.5 ± 0.9* | 150* |
| | 400 | 5.0* | 5.2 ± 0.5* | 71* |
| Mutein III | 250 | 7.0 | 7.2 ± 0.8 | 100 |
| | 400 | 8.5 | 8.7 ± 1.1 | 121 |
| Mutein V | 250 | 7.0 | 7.0 ± 0.6 | 100 |
| | 400 | 9.0* | 9.2 ± 1.2* | 129* |
| Mutein VI | 250 | 7.5 | 7.4 ± 0.9 | 107 |
| | 400 | 9.0* | 9.1 ± 0.9* | 129* |

[a] Treatment, once a day, in eight i.p. injections or less if the survival was shorter.
[b] PBS, phosphate buffered saline given once a day, in eight i.p. injections or less if the survival was shorter.
[c] MST, median survival time. Six mice were used per group.
[d] Mean values and standard deviations.
[e] $T/C, \frac{\text{MST of the treated group}}{\text{MST of the control group}} \times 100\ (\%)$
* Statistical significance compared with the control group.

**Table 3.** The influence of TNF-α and its muteins, administered every 48 h, on the survival time of mice with leukaemia L1210

| Therapeutics[a] | | MST[c] (days) | $\bar{X} \pm SD$[d] | T/C[e] |
|---|---|---|---|---|
| Cytokine | Dose (μg/kg) | | | |
| None | PBS[b] | 7.0 | 6.8 ± 0.5 | |
| TNF-α | 400 | 7.5 | 8.0 ± 1.0 | 107 |
| Mutein III | 400 | 8.5 | 8.8 ± 1.0 | 121 |
| Mutein V | 400 | 9.0* | 9.1 ± 1.3* | 129* |
| Mutein VI | 400 | 9.5* | 9.6 ± 1.1* | 136* |

[a] Treatment, four i.p. injections on days 2, 4, 6, 8 of experiment.
[b] PBS, phosphate buffered saline given in four i.p. injections on days 2, 4, 6, 8 of experiment.
[c] MST, median survival time. Six mice were used per group.
[d] Mean values and standard deviations.
[e] $T/C, \frac{\text{MST of the treated group}}{\text{MST of the control group}} \times 100\ (\%)$
* Statistical significance compared with the control group.

The survival time of leukaemia P388-bearing mice receiving TNF-α or its mutein V, either 250 μg/kg in daily or 400 μg/kg in sequential injections, was almost the same as that of the control group (Tables 4 and 5). Daily injections of 400 μg/kg of these cytokines shortened the lifetime of mice compared with animals from the control group ($p < 0.001$). The lifetime of mice treated with mutein III or VI, at daily doses of 250 or 400 μg/kg, did not differ significantly from that observed in the control group (Table 4). Sequential application of 400 μg/kg of these muteins significantly prolonged the survival time of leukaemia P388-bearing mice in comparison with TNF-α, mutein V or control mice ($0.01 > p > 0.001$). The longest survival time was seen in the group receiving mutein VI in the sequential treatment schedule (Table 5).

**Table 4.** The influence of TNF-α and its muteins, administered daily, on the survival time of mice with leukaemia P388

| Therapeutics[a] | | MST[c] (days) | $\bar{X} \pm$ SD[d] | T/C[e] |
|---|---|---|---|---|
| Cytokine | Dose (μg/kg) | | | |
| None | PBS[b] | 10.0 | 10.5 ± 0.5 | |
| TNF-α | 250 | 11.0 | 11.0 ± 0.6 | 110 |
| | 400 | 8.0 | 8.0 ± 0.9 | 80 |
| Mutein III | 250 | 11.0 | 11.2 ± 0.6 | 110 |
| | 400 | 11.5 | 11.5 ± 0.5 | 115 |
| Mutein V | 250 | 11.0 | 10.9 ± 0.8 | 110 |
| | 400 | 8.0 | 8.0 ± 0.5 | 80 |
| Mutein VI | 250 | 11.5 | 11.6 ± 0.9 | 115 |
| | 400 | 10.0 | 9.8 ± 0.8 | 100 |

[a] Treatment, once a day, in eight i.p. injections or less if the survival was shorter.
[b] PBS, phosphate buffered saline given once a day, in eight i.p. injections or less if the survival was shorter.
[c] MST, median survival time. Six mice were used per group.
[d] Mean values and standard deviations.
[e] T/C, $\frac{\text{MST of the treated group}}{\text{MST of the control group}} \times 100$ (%)
*Statistical significance compared with the control group.

**Table 5.** The influence of TNF-α and its muteins, administered every 48 h, on the survival time of mice with leukaemia P388

| Therapeutics[a] | | MST[c] (days) | $\bar{X} \pm$ SD[d] | T/C[e] |
|---|---|---|---|---|
| Cytokine | Dose (μg/kg) | | | |
| None | PBS[b] | 10.0 | 10.5 ± 0.5 | |
| TNF-α | 400 | 11.0 | 11.2 ± 0.6 | 110 |
| Mutein III | 400 | 13.0* | 13.2 ± 0.5* | 130* |
| Mutein V | 400 | 9.5 | 9.5 ± 0.6 | 95 |
| Mutein VI | 400 | 14.0* | 14.2 ± 1.1* | 140* |

[a] Treatment, four i.p. injections on days 2, 4, 6, 8 of experiment.
[b] PBS, phosphate buffered saline given in four i.p. injections on days 2, 4, 6, 8 of experiment.
[c] MST, median survival time. Six mice were used per group.
[d] Mean values and standard deviations.
[e] T/C, $\frac{\text{MST of the treated group}}{\text{MST of the control group}} \times 100$ (%)
*Statistical significance compared with the control group.

### Discussion

TNF-α is a cytokine which holds strong promise for application in cancer therapy because of its marked antiproliferative effects against various tumour cell lines in both *in vitro* and *in vivo* conditions.[9–15] Originally defined as an antitumoural agent, it is now recognized as a mediator of inflammation and cellular immune responses. The first step in the induction of these activities is the binding of TNF-α to specific cell surface receptors. Two such receptors, termed p55 and p75, have recently been cloned,[16,17] although the TNF-α domain responsible for receptors binding has not been precisely identified. Experiments performed with antibodies generated against amino acids 1–31 of TNF-α support the concept that the receptor-binding domain of TNF-α may be located near the N-terminus of the TNF-α molecule.[7,18,19] In this work we have compared the antitumour effects of TNF-α and its derivatives termed muteins III, V, VI, in which the first 3 to 7 amino acids of native TNF-α have been replaced, against two kinds of murine leukaemias, using two different therapy regimens.

The results of our previous[4,5] and present studies show that submaximally and maximally tolerated doses of TNF-α are those of maximum antitumour effectiveness. Daily administration of this cytokine is more effective than sequential application, although sequential administration of TNF-α seems to be less toxic than this observed in daily protocol. Yet, neither the dose nor the schedule mentioned above had any therapeutic activity in leukaemia P388-bearing mice. Thus, not only dosage, but also duration and sequence of TNF administration, as well as the type of target neoplasia, seem to be of key value in the treatment strategy. An important thing in understanding the mechanisms of antitumour activity of this cytokine is to explain how selectivity is achieved with respect to the diverse TNF-α responses in different cell types and tissues. A clue to understanding this mechanism may come from studies defining the amino acid requirements for the biological activity of TNF-α.[6,7,20–22]

From the data of others it is known that an increase in basicity of the N-terminal segment of TNF-α improves its cytotoxic activity.[7,20] This observation is consistent with our finding that the basic mutein III has more expressed antileukaemic activity than the native molecule of TNF-α. It should be stressed that this mutein binds to both TNF-α receptors, as indicated in *in vitro* studies on human epithelioid carcinoma cells (HeLa cells) or Burkitt lymphoma cells (Jijoye cells). This is in contrast with observations concerning mutein VI, which does not bind to any of TNF-α receptors, either on HeLa or Jijoye cells, and exhibits extremely cytotoxic effect *in vitro*,[7] as well as in our P388 murine leukaemia model. Interestingly, this mutein exhibits very subtle proinflammatory effect and fails to induce endothelial cell responses, including expression of intercellular adhesion molecule-1 (ICAM-1) and secretion of interleukin 6 (IL-6).[6,7] In addition, experimental mice exhibit much better tolerance of high doses of mutein VI compared with native TNF-α, since daily injections of 400 μg/kg of mutein VI significantly prolonged the survival time of mice compared with TNF-α treated group, whose lifetime was even shorter than that observed in control animals. These results suggest that the modification of N-terminal region of TNF-α augments and/or alters mechanisms of antitumour action of the native molecule of TNF-α and allows the minimizing of the side effects of therapy with this cytokine. Mutein V, which *in vitro* appears to exert its effect through an interaction with p75 TNF-α receptor, caused only slight prolongation of life of leukaemia L1210-bearing mice, and no effect against leukaemia P388. This is in agreement with *in vitro* observations, indicating that cytotoxic as well as proinflammatory effects of mutein V represent intermediate level compared with native molecule of TNF-α on the one hand, and mutein III or VI on the other.[6,7]

In the past few years, a number of experimental observations were made that have provided insights into the cytotoxic mechanism of TNF-α action. The question of structure–function relationship to TNF-α activity seems to be an important clue in the understanding of the operation of the TNF-α-signalling system. The results presented in this report and the observations of others indicate that mechanisms of TNF-α cytotoxic action are governed at least in part by the nature of the N-terminal amino acids. Further studies will hopefully yield a cohesive model of how TNF-α shares its biological activities, and will allow more rational transposing of the positive preclinical data into effective clinical treatment regimens.

### References

1. Duncombe AS, Heslop HE, Turner M, Meager A, Priest R, Exley T, Brenner MK. Tumor necrosis factor mediate autocrine growth inhibition in a chronic leukemia. *J Immunol* 1989; **143:** 3828–3834.
2. Murase T, Hotta T, Saito H, Ohno R. Effect of recombinant human tumor necrosis factor on the colony growth of human leukemia progenitor cells and normal hematopoietic progenitors cells. *Blood* 1987; **69:** 467–472.
3. Robak T. Biological properties of cachectin (TNF). *Postępy Hig Med Dośw* 1991; **45:** 5–12.
4. Warzocha K, Robak T. Antileukemic effects of recombinant human tumor necrosis factor alpha (rh-TNFα) with cyclophosphamide or methotrexate on leukemia L1210 and leukemia P388 in mice. *Acta Hematol Pol* 1992; **23:** 55–62.
5. Warzocha K, Robak T, Korycka A, Graczyk J, Pakulska W, Gałązka G, Kłysik J. The influence of recombinant human tumor necrosis factor alpha, singly and in combination with cyclophosphamide or methotrexate, on leukemia L1210 and normal hematopoiesis in mice. *Arch Immunol Ther Exp* 1991; **39:** 587–595.
6. Tchórzewski H, Zeman K, Kantorski J, Paleolog E, Kahan M, Feldmann M, Kwinkowski M, Guga P, Szymańska B, Parniewski P, Wilk A, Jarosz J. The effect of tumor necrosis factor-α (TNF-α) muteins on human neutrophils *in vitro*. *Mediators Inflamm* 1993; **2:** 41–48.
7. Tchórzewski H, Zeman K, Paleolog E, Brennan F, Feldmann M, Kahan M, Guga P, Kwinkowski M, Szymańska B, Jarosz J, Parniewski P, Kocur E. The effects of tumor necrosis factor (TNF) derivatives on TNF receptors. *Cytokine* 1993; **5:** 125–132.
8. Kłysik J, Konarzewska-Zglińska A, Gałązka G, Uznański B, Okruszek A, Guga P, Wilk A, Koziołkiewicz M, Tchórzewski H, Zembala M, Stec JW. Synthesis and expression in *E. coli* of the gene for human tumor necrosis factor (Ha-TNF). *Arch Immunol Ther Exp* 1991; **39:** 349–356.
9. Blick M, Sherwin SA, Rosenblum M, Gutterman J. Phase I study of recombinant tumor necrosis factor in cancer patients. *Cancer Res* 1987; **47:** 2986–2989.
10. Creagan ET, Kovach JS, Moertel CG, Frytal S, Krols LK. A phase I clinical trial of recombinant human tumor necrosis factor. *Cancer* 1988; **62:** 2467–2471.
11. Haranaka K, Satomi N, Sakurai A. Antitumor activity of murine tumor necrosis factor against transplanted murine tumors and heterotransplanted human tumors in nude mice. *Int J Cancer* 1984; **36:** 263–267.
12. Manda T, Shimomura K, Mukumoto S, Kobayashi K, Mizota T, Hirai O, Matsumoto S, Oku T, Nishigahi F, Mori J, Kikuchi H. Recombinant human tumor necrosis factor: evidence of an indirect mode of antitumor activity. *Cancer Res* 1987; **47:** 3707–3711.
13. Peetre C, Gulberg U, Nilsson E, Olsson J. Effects of recombinant tumor necrosis factor on proliferation of leukemia and normal hemopoietic cells *in vitro*. *J Clin Invest* 1986; **78:** 1694–1780.
14. Sugarman BJ, Aggarwad BB, Hass PE. Recombinant tumor necrosis factor-alpha: effects on proliferation of normal and transformed cells *in vitro*. *Science* 1985; **230:** 943–945.
15. Takahashi M, Mogi Y, Goto Y, Tsushima N, Takahashi Y, Fujikawa K, Watanabe N, Kohgo Y, Sugiyama S, Niitsu Y. A case of cutaneous T cell lymphoma improved with local administration of tumor necrosis factor. *Cancer Res* 1990; **81:** 949–956.
16. Gray PW, Barrett K, Chantry DH, Turner M, Feldmann M. Cloning of human tumor necrosis factor (TNF) receptor cDNA and expression of recombinant soluble TNF-binding protein. *Proc Natl Acad Sci USA* 1990; **87:** 7380–7384.
17. Scall TJ, Lewis M, Koller KJ, Rice GC, Wong GHW, Gatanaga T, Granger GA, Lentz R, Raab H, Khor WK, Goeddel DV. Molecular cloning of a receptor for human tumor necrosis factor. *Cell* 1990; **61:** 361–370.
18. Goh CR, Porter AG. Structural and functional domains in human tumor necrosis factors. *Protein Eng* 1991; **4:** 386–389.
19. Socher SH, Riemen MW, Martinez D, Friedman A, Tai J, Qintero JC, Garsky V, Oliff A. Antibodies against amino acids 1–15 of tumor necrosis factor block its binding to cell-surface receptor. *Proc Natl Acad Sci USA* 1987; **84:** 8829–8833.
20. Soma GI, Kitahara N, Tsuji Y, Kato M, Oshima H, Gatanaga T, Inagawa H, Noguchi K, Tanabe Y, Mizuno D. Improvement of cytotoxicity of tumor necrosis factor (TNF) by increase in basicity of its N-terminal region. *Biochem Biophys Res Commun* 1987; **148:** 629–635.
21. Utsuni T, Hung MC, Klostergaard J. The role of amino functions in recombinant human tumor necrosis factor in expression of biological activity. *Molec Immunol* 1992; **29:** 77–81.
22. Yamagushi J, Kawashima H, Matsuo N. Mutational analysis of structure–activity relationships in human tumor necrosis factor-alpha. *Protein Eng* 1990; **3:** 713–719.

ACKNOWLEDGEMENTS. TNFα and its muteins' syntheses and biochemical analyses were performed in the Department of Bioorganic Chemistry, Łódź, Poland. Production of TNF muteins were supported by the Committee for Scientific Research, Grant No. 662889203 to M.K. and B.S.

**Received 16 May 1994; accepted in revised form 5 July 1994**
